# Association between hs-CRP/HDL with type 2 diabetes mellitus in middle-aged and elderly people: a cross-sectional study from CHARLS

**DOI:** 10.3389/fendo.2025.1471292

**Published:** 2025-03-07

**Authors:** Yan Jiang, Jiali Yu

**Affiliations:** ^1^ Department of Cardiology, Affiliated Hospital of Nantong University, Nantong, China; ^2^ Department of Emergency, Affiliated Hospital of Nantong University, Nantong, China

**Keywords:** type 2 diabetes mellitus, Hs-CRP, HDL, inflammatory markers, CHARLS

## Abstract

**Background:**

Research into the relationship between the ratio of high-sensitivity C-reactive protein (hs-CRP) and high-density lipoprotein cholesterol (HDL-C) concerning type 2 diabetes mellitus (T2DM) is still scarce. The hs-CRP/HDL ratio could be an important biomarker for evaluating the risk of developing diabetes. This study primarily aims to investigate the association between hs-CRP/HDL ratios and the incidence of T2DM within a defined population.

**Methods:**

This analysis was conducted using data from 9,381 participants aged 45 and older, obtained during the 2011 wave of the China Health and Retirement Longitudinal Study (CHARLS). The study evaluated the association between the hs-CRP/HDL ratio and the risk of developing type 2 diabetes mellitus (T2DM) employing multivariate logistic regression, subgroup analyses, smooth curve fitting, and threshold effect analysis.

**Results:**

The overall prevalence of T2DM within the study population was found to be 16.3%, with 46.1% of cases occurring in men and 53.9% in women. Participants diagnosed with T2DM demonstrated a mean hs-CRP/HDL ratio that was 1.2 times higher than that of individuals without diabetes. The adjusted odds ratio (OR) for T2DM associated with hs-CRP/HDL levels was determined to be 0.75 (95% CI: 0.64–0.87). Additionally, a significant interaction was identified between hs-CRP/HDL ratios and variables such as sex and smoking in relation to T2DM risk (P < 0.05). Further subgroup analyses examining factors like age, education, marital status, hukou status, and drinking habits did not reveal any significant interactions (all P values for interaction were >0.05).

**Conclusions:**

The results highlight a robust association between the hs-CRP/HDL ratio and the likelihood of developing T2DM, indicating its potential as a predictive biomarker for the condition. Additional research is required to clarify the relationship between hs-CRP/HDL ratios and the incidence of T2DM.

## Introduction

Type 2 diabetes mellitus (T2DM), responsible for approximately 90–95% of all diabetes mellitus (DM) cases, is a major contributor to global morbidity and mortality (1, 2). Diabetes is a significant risk factor for atherosclerosis and has been linked to a higher likelihood of mortality from various cancers, infections, and cardiovascular conditions (3). The International Diabetes Federation reports that, in 2021, 10.5% of adults aged 20–79 worldwide (equivalent to 536.6 million people) were living with diabetes, a figure projected to rise to 12.2% (643 million) by 2045 (4). Given these concerning statistics, identifying modifiable risk factors for T2DM is essential to improving prevention efforts and optimizing self-management strategies.

Diabetes is often associated with abnormal lipid metabolism, which is a major contributor to the development of diabetic vascular complications. Research indicates a strong association between high low-density lipoprotein cholesterol (LDL-C) levels and a heightened risk of aortic stenosis (5). HDL-C is crucial in lowering cardiovascular risk by enabling reverse cholesterol transport and exhibiting antioxidant, anti-inflammatory, and antithrombotic effects (6). Recent research indicates an inverse relationship between elevated HDL-C levels and the onset of T2DM, especially among middle-aged and older Chinese populations (7). Research by Yang T and colleagues has also identified the triglyceride-to-HDL-C ratio as a critical risk factor for both diabetes and the onset of T2DM (8). Research indicates elevated systemic inflammatory markers in patients with both atherosclerosis and type 2 diabetes (9). This inflammatory state can lead to insulin resistance and adversely affect endothelial function (10). In addition, hs-CRP, a recognised inflammatory biomarker, has been associated with diabetic nephropathy in several studies, highlighting its importance in the prediction and diagnosis of diabetes-related complications (11, 12).

HDL-C and hs-CRP are important and cost-effective biomarkers that are widely used to assess the risk of cardiovascular disease through their ratio (13). Despite well-documented significance in cardiovascular health, research on the hs-CRP/HDL-C ratio’s link to T2DM is limited. This study performed a cross-sectional analysis to explore the significance of the hs-CRP/HDL-C ratio in relation to T2DM. The aim is to improve the understanding of how this biomarker may influence diabetes risk and to support the development of clinical strategies aimed at early detection and intervention.

## Methods

### Study population

**Figure 1:**
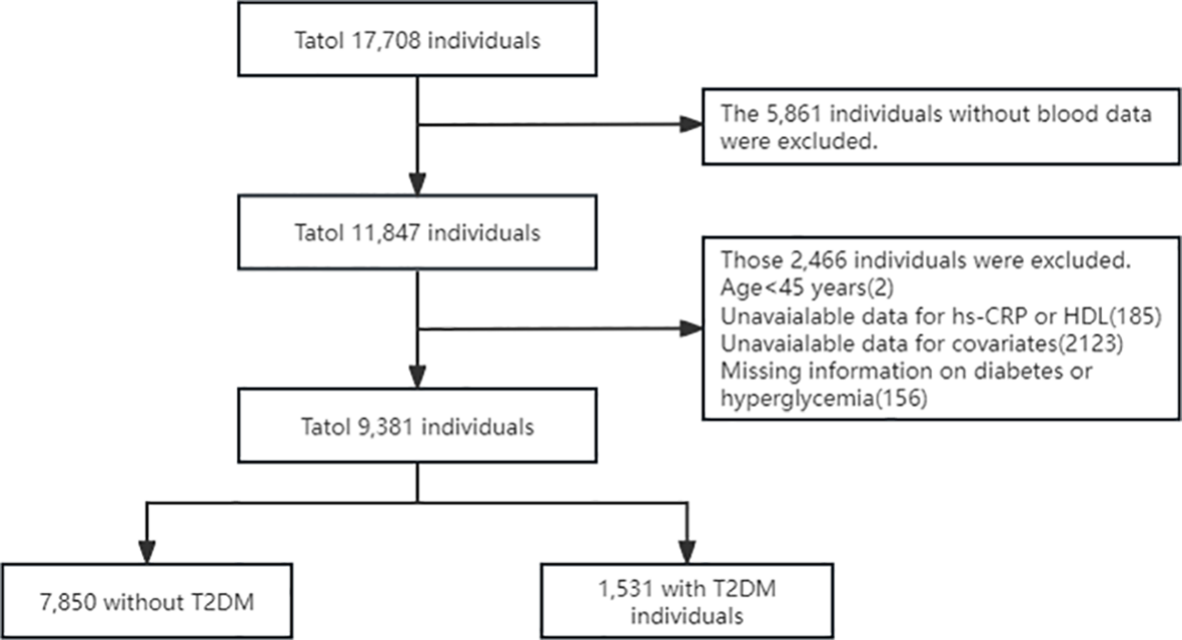
Flow chart of the study.

The CHARLS is an ongoing nationally representative longitudinal survey targeting adults aged 45 years and above. It aims to investigate aging in China and support interdisciplinary research by providing high-quality household microdata. The baseline survey for CHARLS was conducted during 2011–2012, encompassing 450 communities and villages across 150 districts in 28 provinces throughout the country, with subsequent follow-up surveys conducted every 2 to 3 years. Blood samples for the CHARLS dataset were collected during both the baseline and follow-up phases in 2011 and 2015, respectively. The National Development Institute of Peking University (IRB00001052-11015) approved the research project of CHARLS, and all participants signed an informed consent form before participating in the study. This cross-sectional study followed the ‘Strengthening the Reporting of Observational Studies in Epidemiology’ (STROBE) guidelines. All methods were conducted in accordance with relevant regulations and guidelines (14). For this analysis, data were drawn from the 2011 baseline survey, which included an initial cohort of 17,707 participants. Participants were excluded if they were under 45 years of age, missing data on high-sensitivity C-reactive protein (hs-CRP), high-density lipoprotein cholesterol (HDL-C), covariates and lacking documentation of diabetes or hyperglycemia. Finally, we included 9,381 participants in this study. The exclusion process is shown in **Figure 1**.

### Assessment of hs-CRP/HDL-C ratio

The concentrations of high-sensitivity C-reactive protein (hs-CRP) and high-density lipoprotein cholesterol (HDL-C) were measured in refrigerated blood samples using standard enzyme colorimetric assays at the Youanmen Center for Clinical Laboratory, Capital Medical University. The hs-CRP/HDL-C ratio was derived by dividing the hs-CRP concentration (expressed in mg/L) by the HDL-C level (expressed in mg/dL).

### Assessment of type 2 diabetes mellitus

In this study, type 2 diabetes mellitus (T2DM) was defined based on criteria established by the American Diabetes Association in 2005. Specifically, T2DM was diagnosed if participants had a fasting blood glucose level of 126 mg/dL (7 mmol/L) or higher, a random blood glucose level of 200 mg/dL (11.1 mmol/L) or greater, an HbA1c level of 6.5% or above, or if they self-reported a diagnosis of diabetes or hyperglycemia in response to the question, “Have you ever been diagnosed with diabetes or hyperglycemia?”.

### Assessment of covariates

Socio-demographic information included age, sex, education level (divided into ‘primary school or less’ and ‘secondary school or higher’), hukou status (categorized as ‘agricultural’ or ‘non-agricultural’), and marital status (designated as ‘married’ or ‘unmarried’).

Health-related behaviors were assessed through various parameters, including smoking status (categorized as “never” versus “former or current smoker”), drinking patterns (classified as “never,” “former,” or “current drinker”), and duration of sleep. This health-related information was gathered using self-reported questionnaires facilitated by trained interviewers. For anthropometric data, systolic blood pressure (SBP) and diastolic blood pressure (DBP) were measured as the average of three readings obtained with an Omron HEM-7200 sphygmomanometer, ensuring precision in the evaluation of these vital signs.

### Statistical analysis

Continuous variables were presented as means with standard deviations (SD) for normally distributed data and as medians with interquartile ranges (IQR) for skewed distributions. Categorical variables were reported as frequencies and percentages. One-way ANOVA, Kruskal–Wallis H test, or chi-square test was employed to compare the differences of variables among different quartiles the hs-CRP/HDL-C ratio. Logistic regression analyses, incorporating both unadjusted and adjusted models, were employed to examine the relationship between the hs-CRP/HDL ratio and the risk of T2DM development. The adjusted models incorporated various covariates such as age, sex, education level, marital status, hukou status, lifestyle factors like smoking and drinking, body mass index (BMI), SBP, DBP, lipid profile measures including total cholesterol (TC), triglycerides (TG), and low-density lipoprotein cholesterol (LDL-C). Penalized splines were employed for smooth curve fitting to evaluate the association between the hs-CRP/HDL-C ratio and T2DM. Sensitivity analyses were conducted to verify the robustness of the findings, and the trend significance was assessed by categorizing the hs-CRP/HDL-C ratio. Subgroup analyses were performed by stratifying relevant covariates to explore potential effect modification. Data analysis utilized R version 4.2.1 (The R Foundation, Vienna, Austria) and Free Statistics software version 1.9.2 (Beijing Free Clinical Medical Technology Co., Ltd, Beijing, China). *p* < 0.05 indicated statistical significance.

## Results

### Baseline characteristics

**Table 1** outlines the baseline demographic and clinical characteristics of the study population, which included 9,381 participants. The median age of the entire cohort was 71 years (interquartile range [IQR]: 65-78). Notably, individuals diagnosed with type 2 diabetes mellitus (T2DM) were significantly older, with a median age of 73 years (IQR: 67-80), compared to 71 years (IQR: 64-78) for those without diabetes (p < 0.001). While the overall sex distribution was similar, with 46.1% of participants being male, significant differences were observed in clinical parameters. T2DM patients had higher mean systolic blood pressure (135.1 mmHg) compared to Non-T2DM individuals (129.5 mmHg, p < 0.001), as well as higher diastolic blood pressure (77.3 mmHg vs. 75.4 mmHg, p < 0.001).

**Table 1:** Baseline characteristics of patients.

Variables Reaction	Total (n = 9381)	T2DM (n = 1531)	Non-T2DM (n = 7850)	p
Age, Median (IQR)	71 (65, 78)	73(67, 80)	71 (64, 78)	< 0.001
Sex, n (%)				0.646
Male	4321 (46.1)	697 (45.5)	3624 (46.2)	
Female	5060 (53.9)	834 (54.5)	4226 (53.8)	
Education, n (%)				0.792
Primary school or lower	6595 (70.3)	1072 (70)	5523 (70.4)	
Secondary school or higher	2786 (29.7)	459 (30)	2327 (29.6)	
Marital status, n (%)				0.814
Married	8249 (87.9)	1349 (88.1)	6900 (87.9)	
Non-Married	1132 (12.1)	182 (11.9)	950 (12.1)	
HuKou status,n (%)				< 0.001
Agricultrue	7768 (82.8)	1205 (78.7)	6563 (83.6)	
Others	1612 (17.2)	326 (21.3)	1286 (16.4)	
Smoking, n (%)				0.626
Never	3655 (39.0)	588 (38.4)	3067 (39.1)	
Former or current	5726 (61.0)	943 (61.6)	4783 (60.9)	
Drinking, n (%)				0.071
Never	2313 (24.7)	348 (22.7)	1965 (25)	
Former	736 (7.8)	111 (7.3)	625 (8)	
Current	6332 (67.5)	1072 (70)	5260 (67)	
SBP(mmHg), Mean ± SD	130.4 ± 21.5	135.1 ± 21.5	129.5 ± 21.4	< 0.001
DBP(mmHg), Mean ± SD	75.7 ± 12.2	77.3 ± 11.6	75.4 ± 12.3	< 0.001
BMI, Median (IQR)	23.1 (20.8, 25.8)	24.3 (21.8, 27.1)	22.9 (20.7, 25.5)	< 0.001
Glucose (mg/dl), Mean ± SD	110.1 ± 36.7	161.4 ± 66.1	100.1 ± 12.1	< 0.001
TC(mg/dl), Mean ± SD	193.2 ± 38.5	199.6 ± 42.3	192.0 ± 37.6	< 0.001
TG(mg/dl),Median (IQR)	131.4 ± 95.5	185.1 ± 154.8	121.0 ± 74.6	< 0.001
HDL-C (mg/dl), Mean ± SD	51.2 ± 15.2	46.4 ± 15.5	52.1 ± 15.0	< 0.001
LDL-C (mg/dl), Mean ± SD	116.3 ± 35.1	114.4 ± 40.2	116.7 ± 34.0	0.018
hs-CRP (mg/l), Median (IQR)	1.0 (0.6, 2.2)	1.4 (0.7, 3.0)	1.0 (0.5, 2.0)	< 0.001
HbA1c(%), Mean ± SD	5.3 ± 0.8	6.1 ± 1.5	5.1 ± 0.4	< 0.001
hs-CRP/HDL-C*100, Median (IQR)	2.1 (1.0, 4.8)	3.2 (1.5, 7.5)	2.0 (1.0, 4.4)	< 0.001
Quartiles of hs-CRP/HDL-C, n (%)				< 0.001
Q1Notes: data	3127 (33.3)	335 (21.9)	2792 (35.6)	
Q2	3127 (33.3)	484 (31.6)	2643 (33.7)	
Q3	3127 (33.3)	712 (46.5)	2415 (30.8)	

data presented are mean ± SD, median (IQR), or N (%).

T2DM, type 2 diabetes mellitus; SBP, systolic blood pressure; DBP, diastolic blood pressure; BMI, body mass index; TC, total cholesterol; TG, Triglycerides; HDL-C, high-density lipoprotein cholesterol; LDL-C, low-density lipoprotein cholesterol; hs-CRP, Hypersensitive C-reactive protein; HbAc1 glycosylated hemoglobin.

Furthermore, individuals with T2DM presented a higher median body mass index (BMI) of 24.3 compared to 22.9 in the Non-T2DM group (p < 0.001), along with significantly elevated glucose levels (161.4 mg/dL vs. 100.1 mg/dL, p < 0.001). Lipid profile analysis indicated that T2DM patients had notably higher total cholesterol levels (199.6 mg/dL vs. 192.0 mg/dL, p < 0.001) and triglycerides (185.1 mg/dL vs. 121.0 mg/dL, p < 0.001), while exhibiting lower high-density lipoprotein cholesterol (HDL-C) levels (46.4 mg/dL vs. 52.1 mg/dL, p < 0.001). Additionally, levels of hs-CRP were significantly greater in the T2DM cohort (1.4 mg/L) compared to Non-T2DM individuals (1.0 mg/L, p < 0.001). The hs-CRP/HDL-C ratio was also markedly higher in the T2DM group at 3.2, in contrast to 2.0 for the Non-T2DM group (p < 0.001).

### Correlations between the initial hs-CRP/HDL-C ratio and type 2 diabetes mellitus

**Table 2** presents the relationship between the hs-CRP/HDL-C ratio and the likelihood of developing type 2 diabetes mellitus (T2DM), including the analysis of different quartiles of this ratio. There was a clear trend indicating that the risk of T2DM increased progressively across the quartiles of the hs-CRP/HDL-C ratio (p for trend <0.001). Specifically, when comparing the first quartile (Q1) to the second quartile (Q2), the latter exhibited the highest risk for T2DM, with an odds ratio (OR) of 0.75 (95% CI: 0.64–0.87) after controlling for confounding factors such as age, sex, body mass index (BMI), smoking status, and alcohol intake (**Table 3**).

**Table 2:** Univariate analysis of association between factors of T2DM.

Variable	OR_95CI	P_value
Age(years)	0.98 (0.98~0.99)	<0.001
Sex,n(%)		
Male	1	
Female	0.97 (0.87~1.09)	0.646
Education,n(%)		
Primary school or lower	1	
Secondary school or higher	0.98 (0.87~1.11)	0.792
Marital status,n(%)		
Married	1	
Non-Married	1.02 (0.86~1.21)	0.814
HuKou status,n(%)		
Agricultrue	1	
Others	0.72 (0.63~0.83)	<0.001
Smoking,n(%)		
Never	1	
Former or current	0.97 (0.87~1.09)	0.626
Drinking,n(%)		
Never	1	
Former	1 (0.79~1.26)	0.981
Current	0.87 (0.76~0.99)	0.036
SBP(mmHg)	0.99 (0.99~0.99)	<0.001
DBP(mmHg)	0.99 (0.98~0.99)	<0.001
BMI	0.92 (0.91~0.93)	<0.001
Glucose(mg/dl)	0.87 (0.86~0.88)	<0.001
TC(mg/dl)	1 (0.99~1)	<0.001
TG(mg/dl)	0.99 (0.99~0.99)	<0.001
HDL(mg/dl)	1.03 (1.02~1.03)	<0.001
LDL(mg/dl)	1 (1~1)	0.018
hs-CRP(mg/l)	0.99 (0.98~0.99)	<0.001
HbA1c(%)	0.15 (0.13~0.17)	<0.001
hs-CRP/HDL-C	0.49 (0.38~0.63)	<0.001

data presented are ORs and 95% CIs.

OR, odds ratio; CI, confidence intervals; T2DM, type 2 diabetes mellitus; SBP, systolic blood pressure; DBP, diastolic blood pressure; BMI, body mass index; TC, total cholesterol; TG, Triglycerides; HDL-C, high-density lipoprotein cholesterol; LDL-C, low-density lipoprotein cholesterol; hs-CRP, Hypersensitive C-reactive protein; HbAc1 glycosylated hemoglobin.

**Table 3:** Association of hs-CRP/HDL with the risk of T2DM in the CHARLS.

Variable	Model 1	p	Model 2	P	Model 3	P
hs-CRP/HDL	0.49 (0.38~0.63)	<0.001	0.56 (0.44~0.71)	<0.001	0.59 (0.46~0.76)	<0.001
hs-CRP/HDL,Quartile						
Q1	1(Ref)		1(Ref)		1(Ref)	
Q2	0.66 (0.56~0.76)	<0.001	0.75 (0.64~0.87)	<0.001	0.88 (0.75~1.03)	0.113
Q3	0.41 (0.35~0.47)	<0.001	0.51 (0.44~0.59)	<0.001	0.68 (0.58~0.79)	<0.001
Trend.test	0.64 (0.59~0.68)	<0.001	0.71 (0.66~0.76)	<0.001	0.82 (0.75~0.88)	<0.001

Model 1 was crude model. Model 2 was adjusted for age, sex, BMI, smoking status, drinking status. Model 3 was adjusted for age, sex, education, marital status, HuKou status, smoking status, drinking status, BMI, SBP, DBP, TC, TG and LDL-C.

IQR, interquartile range; T2DM, type 2 diabetes mellitus; CHARLS, China Health and Retirement Longitudinal Study; BMI, body mass index; SBP, systolic blood pressure; DBP, diastolic blood pressure; TC, total cholesterol; TG, Triglycerides; HDL-C, high-density lipoprotein cholesterol; LDL-C, low-density lipoprotein cholesterol; hs-CRP, Hypersensitive C-reactive protein.

**Figure 2:**
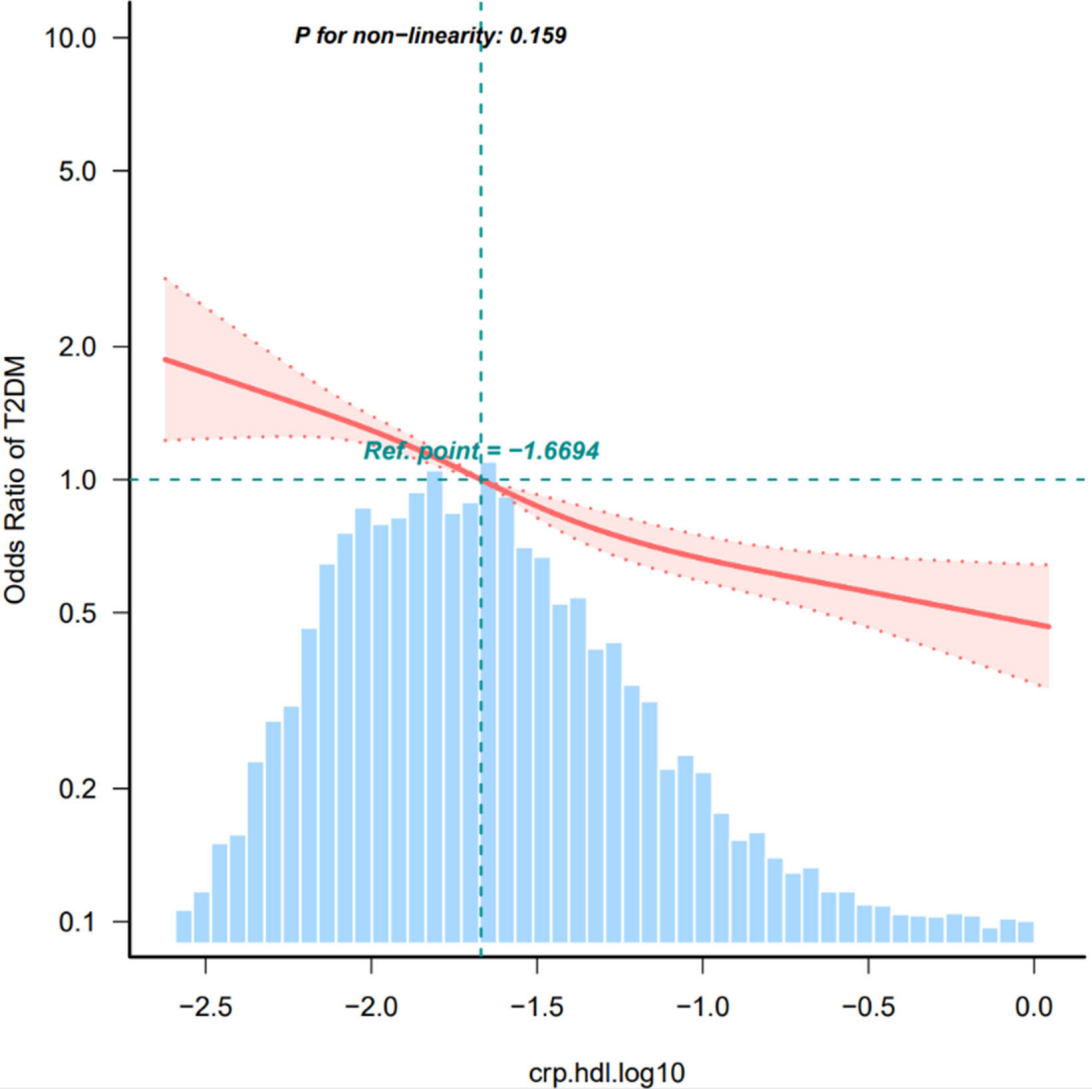
Restricted cubic spline of the association between hs-CRP/HDL and the risk of T2DM. The model was adjusted for age, sex, smoking status, drinking status, BMI. The plot shows a linear relationship between hs-CRP/HDL and the risk of T2DM. T2DM, diabetes mellitus; BMI, body mass index, HDL-C, high-density lipoprotein cholesterol; hs-CRP, Hypersensitive C-reactive protein. Only 99% of the data is shown.

When assessed as a continuous variable, the hs-CRP/HDL-C ratio remained significantly associated with T2DM, yielding an OR of 0.49 (95% CI: 0.38–0.63). **Figure 2** illustrates the association between the hs-CRP/HDL-C ratio and T2DM risk. Additionally, restricted cubic spline regression analysis revealed a linear correlation between the hs-CRP/HDL-C ratio and the likelihood of T2DM (p for linear <0.001). This consistent pattern reinforces the potential of the hs-CRP/HDL-C ratio as an important marker in assessing diabetes risk.

### Stratified analysis

To assess whether the hs-CRP/HDL-C ratio influences the risk of developing type 2 diabetes mellitus (T2DM) differently across various subgroups, participants were categorized based on their individual characteristics. The findings indicated that the impact of the hs-CRP/HDL-C ratio on T2DM risk remained consistent throughout these subgroups. Notably, a significant interaction was observed between the hs-CRP/HDL-C ratio and factors such as sex and smoking status (p for interaction <0.05).

**Figure 3:**
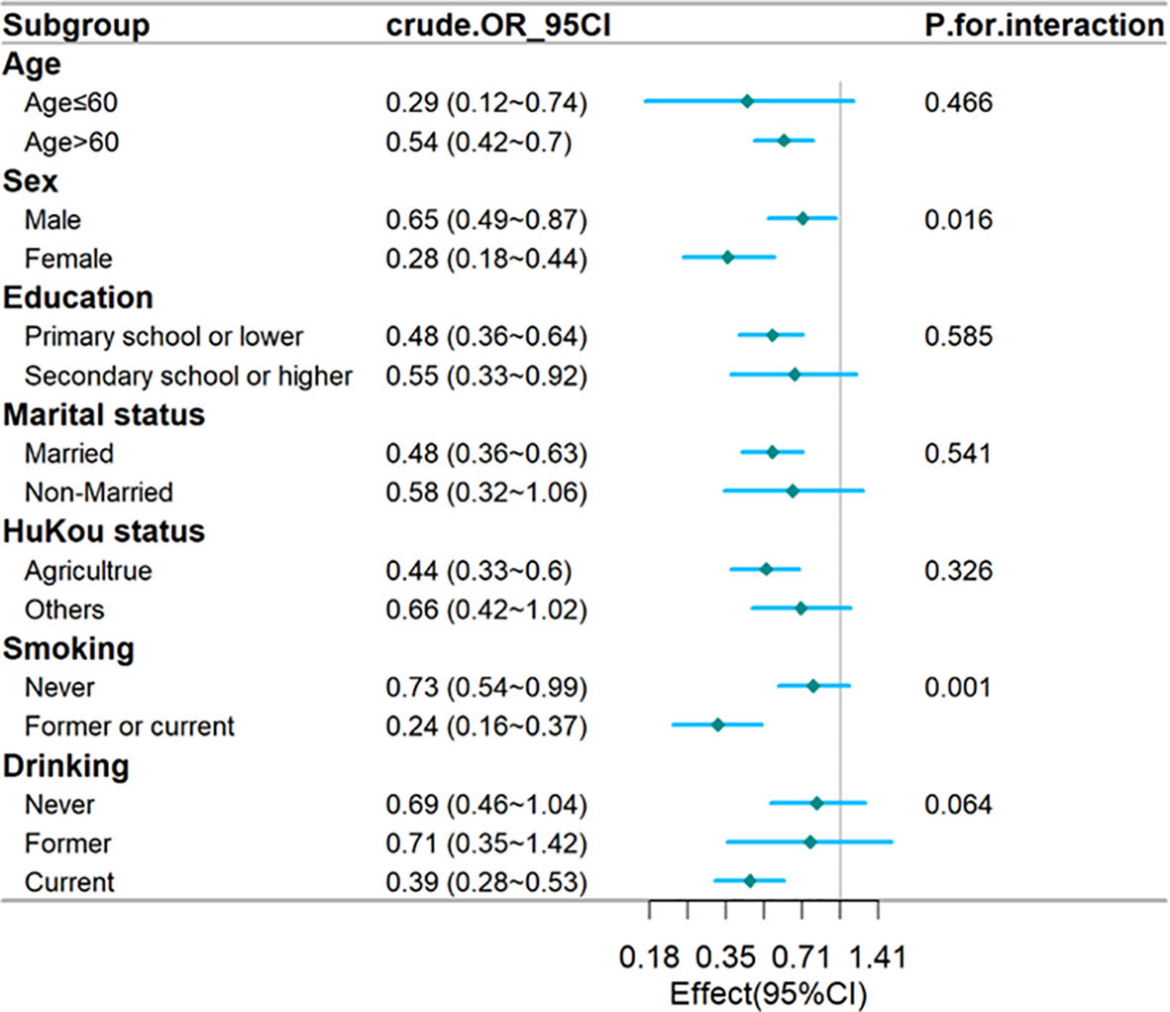
Forest plot of stratified analysis of the association of hs-CRP/HDL-C ratio with the risk of T2DM. The plot shows that there were significant interactions between hs-CRP/HDL-C ratio and sex. OR, odds ratio; CI, confidence intervals; T2DM, type 2 diabetes mellitus; hs-CRP, Hypersensitive C-reactive protein; HDL-C, high-density lipoprotein cholesterol.

In males, an increase of one interquartile range (IQR) in the hs-CRP/HDL-C ratio was linked to a 99.5% increase in the likelihood of developing T2DM, resulting in an odds ratio (OR) of 0.65 (95% CI: 0.49–0.78). However, it is important to consider that these results may lack clinical significance due to the multiple comparisons conducted and the similar patterns observed in the associations (**Figure 3**).

## Discussion

In a cross-sectional study involving 9,381 participants, both baseline and follow-up data revealed a significant link between the development of T2DM and the hs-CRP to HDL-C ratio. Subgroup analyses based on age, education, marital status, hukou status, and drinking habits indicated no significant interactions. A significant interaction was identified between the hs-CRP/HDL-C ratio and variables like sex and smoking status (p < 0.05). The hs-CRP/HDL-C ratio appears to be a significant biomarker for predicting T2DM risk.

To control for potential confounders, we established three logical regression models to analyze the association between hs-CRP/HDL-C ratio and T2DM. In fully adjusted model 3, the effect value is 0.59 (0.46~0.76). Simultaneously, we divided hs-CRP/HDL-C ratio into three groups and conducted sensitivity analysis on results, which showed that the results were stable and reliable (**Table 2**). Additionally, we validated the results in age, education, marital status, hukou status, and drinking habits and found that they were stable in all subgroups without interaction. A significant interaction was identified between the hs-CRP/HDL-C ratio and variables like sex and smoking status (p < 0.05) (**Figure 3**). The fitting curve (**Figure 2**) between hs-CRP/HDL-C ratio and T2DM was drawn after adjustment according to model 2, in order to better present this result and observe the linear relationship between hs-CRP/HDL-C ratio and T2DM. This is also consistent with our validation of trend lines.

Several studies explored the associations between various biomarkers and the risk of T2DM in middle-aged and elderly populations. Hs-CRP was found to be positively associated with T2DM risk (15). The hs-CRP/HDL-C ratio emerged as a significant predictor of cardiovascular disease risk (13). Several lipid parameters, including non-HDL-C, TG, TC/HDL-C, and TG/HDL-C, demonstrated superior performance in predicting T2DM incidence (16, 17). Time-dependent TG/HDL-C ratios were positively associated with T2DM risk in elderly Chinese populations (18). Additionally, hs-CRP levels were linked to metabolic syndrome and low HDL-C levels (19). These findings highlight the importance of considering inflammatory and lipid markers in assessing T2DM risk and potential interventions for middle-aged and elderly individuals.

Some studies may partially explain the underlying mechanisms. Throughout T2DM progression, plasma triglycerides and LDL-C typically rise, while HDL-C levels tend to decline (20). HDL-C exhibits cardioprotective effects, including antioxidant, antithrombotic, and anti-inflammatory properties. It plays a crucial role in reverse cholesterol transport, modulates immune processes, and inhibits endothelial dysfunction (21, 22). HDL-C dysfunction, including structural changes and metabolic abnormalities, is linked to the pathogenesis and prognosis of T2DM, beyond mere alterations in HDL-C levels (23). However, low HDL-C levels may affect glucose homeostasis by reducing insulin secretion, insulin sensitivity and direct glucose uptake by muscle via adenosine monophosphate(AMP)-activated protein kinase (24). In addition, HDL-C has also been shown to potentially regulate glucose homeostasis through mechanisms such as insulin secretion, direct glucose uptake by muscle and increased insulin sensitivity (25). This impacts insulin secretion and blood glucose regulation, potentially leading to diabetes. HDL-C plays a role in cholesterol transport and reduced HDL-C levels can compromise cell membrane stability, impacting insulin receptor structure and function, which in turn influences insulin secretion and sensitivity (26–28). These findings support the idea that raising HDL-C levels could be a viable therapeutic approach to reduce the risk of developing T2DM (29). Hs-CRP, a well-established marker of inflammation, has been recognized as a risk factor for T2DM in cohort studies, particularly when elevated (15). In addition, people in the pre-diabetic stage with moderate to high levels of hs-CRP have an increased risk of developing diabetes compared to those with lower levels (30).

Most research into the prediction of T2DM risk has focused on individual lipid or inflammatory markers. In contrast, this study examines the interaction between lipid metabolism and inflammation, focusing specifically on composite inflammatory lipid markers found in peripheral blood. Such an integrated approach could be of significant benefit to clinicians, particularly primary care providers, by facilitating early intervention in patients at risk of diabetes, ultimately helping to reduce disease progression and improve patient outcomes. Therefore, we speculate that lowering hs-CRP/HDL ratio may be a good way to regulate the health status of T2DM patients. However, more prospective studies are needed to determine whether this study is applicable to a wider population.

Nonetheless, this research is subject to certain limitations. Firstly, it relies on secondary data, which can be affected by measurement inaccuracies, variations in case definitions and differences in study design. The inclusion of participants with baseline hypertension and hyperlipidaemia, along with their use of antihypertensive and lipid-lowering medications, may impact the accuracy of hs-CRP and HDL-C measurements. This study focused on middle-aged and elderly individuals in China, indicating the necessity for future research to validate findings in a more diverse population. Finally, the cross-sectional design limits the ability to establish a temporal relationship between hs-CRP and HDL-C levels.

## Conclusions

The findings from this cross-sectional study indicate a positive association between the hs-CRP/HDL ratio and the risk of developing type 2 diabetes mellitus (T2DM) within the CHARLS cohort. Nonetheless, additional research is needed to validate and confirm these results in different populations and settings.

## Data Availability

The original contributions presented in the study are included in the article/supplementary material, further inquiries can be directed to the corresponding author/s.
